# Cryptic effects of habitat declines: coral-associated fishes avoid coral-seaweed interactions due to visual and chemical cues

**DOI:** 10.1038/srep18842

**Published:** 2016-01-04

**Authors:** Rohan M. Brooker, Simon J. Brandl, Danielle L. Dixson

**Affiliations:** 1 School of Marine Science and Policy, University of Delaware, Lewes, DE 19958, USA; 2 School of Biology, Georgia Institute of Technology, Atlanta, GA 30318, USA; 3 Tennenbaum Marine Observatories Network, Smithsonian Environmental Research Centre, Edgewater, MD, 21037, USA; 4 College of Tropical and Marine Science, James Cook University, Townsville, QLD 4811, Australia; 5 ARC Centre of Excellence for Coral Reef Studies, James Cook University, Townsville, QLD 4811, Australia

## Abstract

Seaweed-dominated coral reefs are becoming increasingly common as environmental conditions shift away from those required by corals and toward those ideal for rampant seaweed growth. How coral-associated organisms respond to seaweed will not only impact their fate following environmental change but potentially also the trajectories of the coral communities on which they rely. However, behavioral responses by coral-associated organisms to seaweeds are poorly understood. This study examined interactions between a guild of obligate and opportunistic coral-feeding butterflyfishes (Chaetodontidae) and scleractinian corals to determine whether fishes continue to interact with corals in contact with seaweed or if they are avoided. Under natural conditions, all species interacted almost exclusively with seaweed-free corals. In a controlled patch reef experiment, fishes avoided corals in physical contact with seaweed, irrespective of dietary preferences. When visual seaweed cues were removed, butterflyfish continued to avoid corals that had been in contact with the allelopathic *Galaxaura filamentosa*, suggesting that chemical cues produced by coral-seaweed interactions are repellent. These findings suggest that, due to deleterious visual and chemical cues produced by coral-seaweed interactions, coral-associated organisms may struggle to locate resources as seaweed-free corals decline in abundance.

In many ecosystems, biotic organisms fulfill an essential role as ecosystem engineers, creating structural complexity, providing essential resources, and promoting biodiversity^1^. For instance, trees create a complex array of habitats and resources that are exploited by whole communities of plant, animal, and fungal species^2^. As many species depend on the resources these ecosystem engineers provide, their abundance and condition can have a strong effect on the health of associated communities. Likewise, how associated species exploit the resources provided may influence the distribution, abundance and condition of ecosystem engineers. Understanding how habitat-building organisms interact with the species that use them is increasingly essential given a rise in impacts that threaten to alter habitat composition. Climate change and other direct anthropogenic effects are rapidly changing the ecology of many habitats by causing shifts in the relative abundance of key habitat-building organisms. For example, increasing herbivore abundance and rising sea surface temperatures threaten many temperate kelp forests^3^, while numerous terrestrial forest ecosystems are being cleared in favor of agriculture and urban expansion^4^,^5^. The consequences of this will not only impact the availability of certain habitats and resources but will also increase pressure on resources that remain.

A dramatic example of how anthropogenic effects can alter habitat community composition can be seen in the various coral reef systems worldwide that have shifted from coral-dominated to seaweed-dominated benthic communities^6^. Seaweed (i.e. macroalgae) in itself is a natural, and functionally important, component of healthy coral reef systems^7^. However, under the stable ecological conditions favorable to coral development, seaweed growth is generally suppressed due to foraging by herbivorous fishes *sensu lato*^8^,^9^ along with nutrient limitation^10^,^11^, allowing corals to dominate benthic substrates. However, various recent anthropogenic impacts, such as the overharvesting of herbivorous fishes^12^ and increasing coastal eutrophication^10^, have created conditions on many reefs favorable to rampant seaweed growth. Increases in seaweed dominance of substratum can not only limit the expansion of established coral colonies^13^,^14^, but can also limit the availability of surfaces suitable for larval settlement and development^15^,^16^, preventing the replenishment of coral communities. Interactions between corals and seaweed can also negatively affect the condition and fitness of colonies. For instance, shading can reduce rates of photosynthesis, abrasion can result in tissue damage at contact points, and attenuation of sediments and associated microbial communities can increase rates of coral disease^17^. Contact with some seaweeds can also lead to tissue mortality due to the allelopathic chemicals within the thalli^18^. Thus, shifts from coral- to seaweed-dominated benthic communities will have dramatic flow-on effects for reef associated organisms, such as fishes, many of which rely on scleractinian corals for survival.

While the habitat requirements of coral reef fishes are highly variable, the abundance and distributions of many species are closely related with the spatial structure of reef substrata, particularly scleractinian coral abundance^19^, structural complexity^20^,^21^ and overall community composition^22^,^23^. Such associations arise as these corals provide essential shelter^24^, access to food resources^25^, and recruitment cues^26^ for many fishes. Butterflyfishes (f. Chaetodontidae), are a ubiquitous and much studied component of coral reef communities worldwide^27^. While these fishes exhibit a variety of dietary strategies^28^, they are best known for the high degree of opportunistic and obligate corallivory found within this family, especially within the genus *Chaetodon*^25^,^29^. Not surprisingly, associations between these butterflyfishes and scleractinian corals are often particularly strong^30^, with significant declines in abundance observed following the loss of scleractinian corals^31^,^32^. Many corallivorous butterflyfishes are highly selective between available prey^33^,^34^. Therefore, given their abundance and direct or indirect reliance on corals for food, the foraging patterns and behavior of these fishes may have some influence on the fitness and distribution of corals within their habitats^35^.

Understanding these patterns is vital given global declines in coral cover and increases in seaweed abundance. However, the responses of butterflyfish to seaweed, in particular whether seaweed affects resource selection, are poorly understood. Consequently, the objectives of this study were to investigate how obligate and opportunistic coral-feeding butterflyfishes associate with corals. Specifically, we determined 1) whether fishes distinguish between corals in contact with, or free of, seaweed and if they do 2) if they are attracted to, or avoid coral-seaweed interactions. If coral-seaweed interactions were avoided, we determined 3) whether this response was due to the visual or chemical cues produced by the interaction and 4) whether responses differed depending on the seaweed species or the degree of reliance on scleractinian food sources.

## Results

### Survey of butterflyfish-coral-seaweed associations

**Figure 1:**
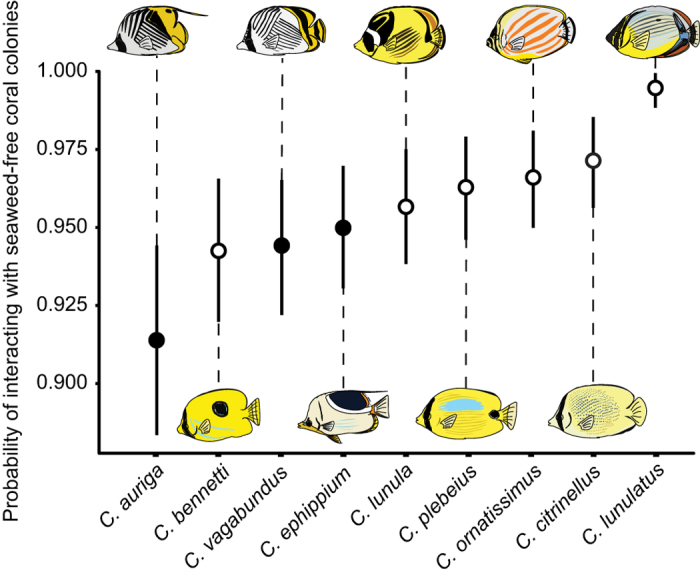
Interspecific differences in the probability that a butterflyfish will interact with seaweed-free corals. Plotted values (circles) represent the predicted mean values from a Bayesian mixed model for each species with the 95% credible interval marked by the line. Predicted values were obtained by marginalizing over the random effects of focal individuals. Given the different degree to which butterflyfishes rely on live coral, group affiliations were obtained from the literature and fitted to the species, with opportunistic coral feeders marked by filled circles, while obligate corallivores are marked by open circles.

Overall, of the 5,391 total associations between butterflyfish and coral recorded during the present survey, 96% of these were with corals that were not in contact with seaweed. However, only one species, *C. lunulatus*, did not associate with any corals in contact with seaweed throughout the survey and there was some variation in the degree of acceptance (Fig. 1, Supplementary table S1 online). *C. auriga* showed the highest rate of interaction with corals in contact with seaweed (BMM: *posterior mean* (*PM*) = 2.94; *95% credible interval* (*CI*) = 2.47 − 3.45), followed by *C. bennetti* (*PM* = 3.49, *CI* = 2.93 − 4.05), *C. vagabundus* (*PM* = 3.53, *CI* = 3.03 − 4.09), and *C. ephippium* (*PM* = 3.67, *CI* = 3.13 − 4.21). In contrast, the lowest rate of interaction was exhibited by *C. lunulatus* (*PM* = 6.73, *CI* = 5.08 − 8.23), followed by *C. citrinellus* (*PM* = 4.41, *CI* = 3.73 − 5.08), *C. ornatissimus* (*PM* = 4.18, *CI* = 3.59 − 4.76)*, C. plebeius* (*PM* = 4.06, *CI* = 3.49 − 4.68), and *C. lunula* (*PM* = 3.86, *CI* = 3.30 − 4.21).

### Patch-reef experiment

**Figure 2:**
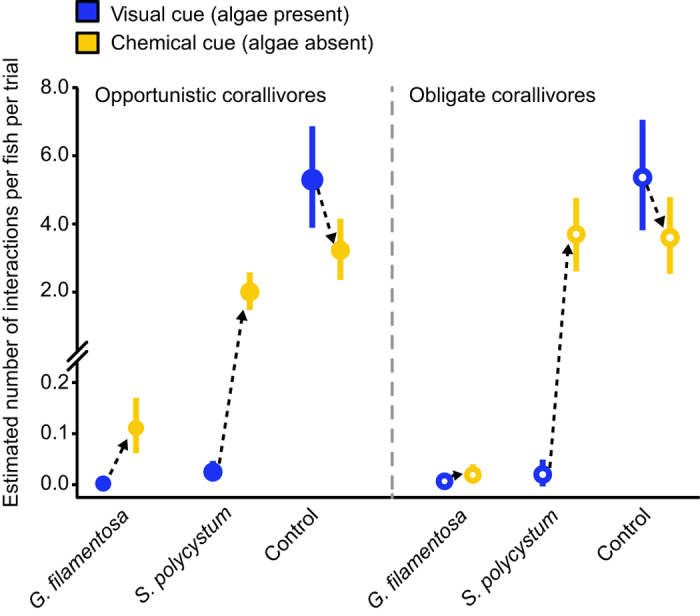
The response of butterflyfishes to manipulative addition of two different algae to coral colonies in a patch-reef experiment. Both obligate (empty circles) and opportunistic (filled circles) corallivores strongly avoid coral colonies associated with algae when visual cues are presented. However, if the algae are removed and only chemical traces of the algae are present, both groups respond positively to the removal of *S. polycystum*, while only opportunistic corallivores show a slight positive response to the removal of *G. filamentosa*. The plotted values represent the predicted values obtained from a Bayesian mixed model and divided by the number of individual fishes present during each trial. Values were marginalized over the random effects of the patch-reef set, which was used longitudinally for trials with or without the algae present.

When offered equal choices between patch reefs with *Sargassum polycystum*, *Galaxaura filamentosa* and control reefs without seaweed, the experimental treatment, the cue provided, and the extent of dietary reliance on live coral (obligate vs. opportunistic corallivore) significantly influenced the number of interactions butterflyfishes exhibited during trials (Fig. 2). In the initial stage of the experiment where seaweed was visually present, all butterflyfish species surveyed preferred to interact almost exclusively with the control patch reefs, showing an overwhelming aversion towards coral patch reefs with attached seaweed. However, after removing the seaweed and relocating the patch reefs, therefore providing a new set of fishes with only a chemical cue of previous seaweed presence, the effects of the two seaweed treatments differed markedly in their effect on the two groups of fishes, consistently eliciting a strong positive response in the *S. polycystum* treatment, while the removal of *G. filamentosa* had weak or no effects. For obligate coral feeders, patch reefs previously associated with *G. filamentosa* still had a negative effect on the foraging behavior of butterflyfishes, while previous associations with *S. polycystum* positively affected the fishes’ choice to interact with the patch reefs when no visual cue was present. Although broadly similar, opportunistic corallivores showed a slight increase in the acceptance of patch reefs previously associated with *G. filamentosa* when the seaweed was removed; opportunistic corallivores showed the same strong positive response to seaweed removal in the *S. polycystum* treatment (Table 1).

## Discussion

The presence of seaweed appears to have a substantial impact on patterns of resource use by butterflyfishes, with both obligate and opportunistic coral-feeding species actively avoiding corals in contact with seaweed under natural and experimental conditions. The visual presence of seaweed may act as a deterrent to butterflyfishes, indicating that a coral potentially has a reduced nutritional value due to the energetic costs related with stress. Seaweed may also physically impede access to food resources such as coral polyps or coral-associated invertebrates. Species-level variation in the levels of association between seaweed-associated and seaweed-free corals may reflect cryptic differences in dietary preferences or other factors such as morphology. For instance, the opportunistic species *C. auriga* may associate prey abundance with factors such as structural complexity produced by seaweeds, while morphological constraints^36^ may restrict the obligate species *C. lunulatus* to foraging on seaweed-free corals. Overall, associations broadly aligned with diet as, with the exception of *C. bennetti*, all obligate species displayed a higher chance of interacting with seaweed-free corals.

Interestingly, avoidance not only reflected the physical presence of seaweed but also occurred when seaweed was removed, with all butterflyfishes continuing to largely bypass corals that had been in contact with an allelopathic seaweed species, *G. filamentosa.* In contrast with treatment patch reefs, the number of interactions with control patch reefs declined for both obligate and opportunistic species when seaweeds were removed. It is possible that the increased availability of attractive foraging grounds (i.e. corals that had been in contact with *S. polycystum*) reduced foraging pressure on control corals. Overall, the results of this study suggest that both visual and chemosensory systems are employed by butterflyfishes during habitat interactions and when selecting resources, and that the multisensory cues produced as a result of coral-seaweed interactions have the potential to repel butterflyfishes. In particular, it appears that the chemical signature of corals may be altered by interactions with allelopathic seaweeds either as a result of stress, persistent seaweed chemicals, or some form of chemical defense produced by the coral itself.

The overwhelming avoidance of corals that had been in contact with *G. filamentosa* may reflect the highly toxic nature of this alga to scleractinian corals. At least six lipid soluble allelopathic metabolites are produced within the surface layer of the *G. filamentosa* thallus^18^; with exposure to these compounds causing reduced photosynthetic activity in corals, and possible bleaching or mortality at the contact site. In contrast, exposure to extracts from *S. polycystum* do not induce these same detrimental effects, nor does shading or abrasion caused by contact with *S. polycystum* thalli^18^. In addition, it has recently been shown that sustained multi-day competition between *G. filamentosa* and corals can stimulate increased allelopathic compound concentrations within the thallus, leading to greater coral tissue damage^37^. This same study indicated that, as allelopathic chemical concentrations increased, seaweed became more palatable to herbivorous fishes. This suggests that the allelochemicals themselves are not repellant to non-herbivorous fishes, with continued avoidance likely due to the odor of damaged or stressed coral tissue at interaction points rather than residual traces of chemicals on or around the coral. Relatively little is known regarding defenses by the coral holobiont to seaweed competition, but responses may be limited to mechanical defenses such as the expulsion of nematocysts^38^.

While it is apparent that the presence of seaweed makes corals unattractive to butterflyfishes, the underlying reasons for this are unclear. While food selection in fishes is often influenced by the need to avoid predators while feeding^39^, adult butterflyfishes are relatively immune to predation^40^,^41^, and it is likely that coral-seaweed interactions are avoided due to the effect of seaweed on coral dietary quality. Coral tissue is a comparatively low-quality food source^42^ meaning corallivores must often forage constantly to meet energetic requirements^43^, and will differentiate between available prey based on small differences that affect foraging efficiency^44^. The presence of seaweed could reduce coral prey quality and foraging efficiency relative to unaffected corals in a variety of ways. Recent evidence has shown that contact with allelopathic seaweeds can have an almost immediate negative effect on coral tissue condition at contact points, with the affected area increasing in size with time^45^. Over longer time frames, shading due to overgrowth could reduce photosynthetic activity^46^, leading to depletion of energetic reserves. Tissue damage caused by abrasion, allelochemicals, or disease will also require the diversion of energy towards defense and repair. Seaweed may also alter the abundance or quality of coral-associated organisms preyed on by opportunistic corallivores. For instance, seaweed competition has a negative effect on the condition of other sessile invertebrates such as sponges^47^, while the abundance of mobile coral-associated invertebrates can decline as corals become stressed^48^. Additionally, the physical presence of seaweed may make it harder to visually or chemically locate coral polyps or coral-associated prey, or harder to access prey when detected. Seaweed visual and chemical cues may therefore signal to butterflyfish that these corals should be avoided. Therefore, the deleterious effects of seaweed contact may be offset somewhat by reductions in the energetic costs of fish predation. While both opportunistic and obligate corallivorous butterflyfishes avoid coral-seaweed interactions, species-level effects of seaweed on foraging were not determined. Further work that examines inter-specific variation in behavioral responses to seaweeds may reveal subtle differences between species.

Regardless of the underlying causes for the general avoidance of coral-seaweed interactions, it is likely that these patterns could have implications for the broader reef community given increasing seaweed dominance, as well as a multitude of other anthropogenic disturbances that increase the risk of coral mortality, and feedback mechanisms that prevent population recovery. This makes the persistence of remaining healthy coral colonies increasingly critical within degrading systems as these colonies become a primary source of new coral larvae, vital habitat and food for a multitude of organisms^25^,^49^, and the source of key settlement cues^26^. Corallivorous fishes that remove tissue without damaging the underlying skeleton are often assumed to have minimal ecological impact on coral communities^50^. However, recent studies have shown that each adult butterflyfish can remove up to 3 g of coral tissue per day^35^, with butterflyfish communities consuming up to 13.5% of the total available coral biomass, and 79% of preferred coral productivity annually^51^. Declines in tissue biomass also directly reflect grazing intensity^52^. If butterflyfish communities on seaweed-dominated reefs forage exclusively on seaweed-free colonies, chronic concentrated predation may lead to a variety of sub-lethal effects such as increased energetic expenditure on repair, reduced growth, lower overall colony fitness, and increased susceptibility of seaweed-free colonies to disturbance effects.

Increased seaweed abundance could also have deleterious outcomes for resident butterflyfish if the availability of unimpeded corals diminishes. Sub-lethal declines in condition and fitness potential may occur if fishes devote a greater proportion of time towards searching for seaweed-free corals, or must maintain and defend larger territories from conspecifics as a result, increasing overall energetic demands. Alternatively, if fishes continue to feed on corals impeded by seaweed, this could also negatively affect condition if the nutritional quality of these corals is reduced. Coral-seaweed interactions may also affect recruitment if resident butterflyfishes exhibit reduced reproductive fitness, or if negative sensory cues also repel larvae^26^. However, given the limited temporal scale of this study, it remains unclear whether butterflyfishes will continue to avoid coral-seaweed interactions over longer time frames. While this study was limited to butterflyfishes at one location, there is substantial behavioral congruence between coral-feeding and coral-associated animals with regards to coral preferences^25^,^49^, and avoidance of coral-seaweed interactions may prove to be a taxonomically widespread occurrence. While behavioral studies of responses to seaweed on coral reefs have typically examined herbivorous species, this study shows that even species that would otherwise not interact with seaweeds will be affected by its presence suggesting seaweeds may be a greater threat to reef biodiversity than realized.

## Methods

### Survey of butterflyfish-coral associations interactions

Interactions between nine species of butterflyfishes and scleractinian corals were surveyed at Votua Reef, Viti Levu, Fiji (18° 13.049′S, 177° 42.968′E) in July, 2013. Twenty individual butterflyfishes from each species were followed on snorkel for a 10 minute period, at a minimum distance of three meters. Observational periods began when fish were observed foraging, taken as indication of acclimation to the observer’s presence. During each observation period, individual’s species and their interactions with coral colonies were recorded, noting whether or not the targeted coral was in contact with seaweed (i.e. all macroalgae spp.). Visual assessments suggested corals in contact with macroalgae and those that were macroalgae free occurred in approximately equal frequency. The proportion of benthos covered by scleractinian corals and seaweeds is highly variable across Votua Reef, ranging from ~4–56% coral and 2.4–91% seaweed^53^. For each focal individual only one interaction was recorded on each colony. An interaction was deemed to have occurred when a fish paused from actively swimming and displayed a head-down behavior within the immediate vicinity of a coral as visual confirmation of *de facto* capture of prey was not reliably observable for all interactions. Butterflyfish species were *Chaetodon auriga*, *C. ephippium*, *C. vagabundus* (opportunistic corallivores), and *C. bennetti*, *C. citrinellus, C. lunula, C. lunulatus*, *C. ornatissmus*, *C. plebeius* (obligate corallivores)^54^. Species were assigned into two groups based on the proportion of coral-based prey items found in the stomach contents in previous quantitative studies (<5% coral-based prey = opportunistic corallivore; >50% coral based prey = obligate corallivore, with no species falling between 5–50%)^30^,^54^,^55^.

### Patch-reef experiment

To investigate whether the frequency of butterflyfish-coral interactions reflected the presence or absence of seaweed, a manipulative patch reef experiment was conducted on Votua Reef. Eight sets of six small patch reefs were constructed in open areas, adjacent to reef structure that multiple species of butterflyfish were observed utilizing. Each individual patch reef consisted of a coral rubble base and two pieces of five coral species of an approximately equal size resulting in a 40 cm^3^ patch reef. The corals used were *Acropora formosa, Pocillopora damicornis, Porites cylindrica, Merulina scabricula* and *Montipora digitata*, chosen due to their abundance within the Votua Reef system and relative association with the butterflyfishes during initial surveys. Within each patch reef set, individual reefs were separated by 1 meter from one another. Each patch reef set consisted of three treatments, with two patch reefs per treatment. Different treatments were identified using red, pink, or green flagging tape. To account for any color preference or avoidance by fish this experiment was replicated three times with flagging tape colors rotated amongst treatments. By grouping six patch reefs together in one location, rotating the treatments within the location and replicating the location of patch reef sets in 8 different areas throughout the reef system local spatial and temporal variation was minimized, an important consideration as butterflyfish are known to be territorial.

The impact that the presence of seaweed had on the butterflyfish species/ patch reef treatment frequency of association was assessed using underwater video cameras (GoPro, Woodman Labs). All 48 patch reefs were constructed during low tide on the same day. The following day, two of the six patch reefs in each set had *S. polycystum* (100 g) placed in contact with all coral fragments. Another two patch reefs had *G. filamentosa* (100 g) placed in contact with all coral fragments, while the final two patch reefs were left as a control with no seaweed contact. To initiate coral-seaweed contact, seaweed was secured in place directly next to the coral using cable ties, with cable ties also attached to the control patch reefs. Cable ties were secured to the base of corals ensuring that they did not contact live tissue. In this fashion, the coral-seaweed interaction was maintained while preventing physical damage to either the seaweed or coral. Full plants were used to ensure seaweed did not deteriorate over the trial period. Patch reefs were checked daily to ensure seaweed had not been removed due to wave action or herbivory, although no seaweed was removed during the experimental period. In an effort to limit any effect of diver disturbance, camera stations were constructed at each patch reef set at the same time as seaweed attachment using a dive weight, cable ties and a GoPro mount adapter. The following morning during the rising tide, cameras were quickly placed at the camera stations on snorkel and recording was started.

Cameras were left to record for three hours, and were collected from the reef in the afternoon at low tide. Seaweed was left in contact with the corals for seven days in total. On the seventh day of contact all seaweed was removed from the patch reefs and the entire set up for each patch reef set was relocated to a new location >100 meters from the initial site. On the eighth day post initial seaweed contact, butterflyfish interactions with patch reefs were reassessed using the same methods described for the initial period. During the relocation, the individual patch reefs as well as the patch reef sets were maintained. This relocation was necessary to ensure that the video data with seaweed visually present and the video data without the visual seaweed cues were independent from one another as an entirely different group of butterflyfish would be exposed to the patch reefs following relocation. Using this experimental design, the influence that seaweed has on coral-butterflyfish interactions could be assessed both with and without the visual cues. The initial video data includes the visual seaweed cue although this seaweed was in contact with coral for less than 24 hours. Data collected from the second video analysis did not include the visual cue although recent evidence suggests that chemical cues that identify allelopathic interactions may continue to be emitted by the coral following seaweed removal^45^. Once again, this entire experiment was replicated 3 times to ensure that the patch reef treatment placement and color of flagging tape were not influencing factors.

During each video, every butterflyfish that entered the patch reef set and interacted with the coral was recorded along with its species and the reef treatment it interacted with. As video footage was not at a high enough resolution to determine if fish were consuming coral during interactions bite rates were not recorded. However, as in the survey, an interaction was considered to have occurred when a fish displayed a head down behavior within the immediate vicinity of a patch reef.

### Statistical treatment

To assess species-specific differences in the extent to which butterflyfishes forage on coral colonies in contact with or without seaweed, we used Markov Chain Monte Carlo (MCMC) estimations of a Bayesian mixed effects logistic regression model^56^,^57^. We fitted an individual’s choice for or against a coral colony with or without seaweed respectively as a binary response variable and species affiliation as a fixed effect, while specifying individual animals to have a random effect. This approach was chosen due to cases of complete and near complete separation in the dataset (i.e. perfect prediction of a variable by a predictor), which can lead to non-identifiability of regression models. To forego this issue, we specified weakly informative Cauchy distributed priors for the fixed effects^58^. We further fitted non-informative priors with an inverse Wishart distribution for the random effects and fixed residual variance to the value 1, as residual estimates are unobtainable in binary models. We ran three chains with 3,000,000 iterations, a burn-in of 10,000 iterations and a thinning interval of 1,000. Chain convergence was assessed visually and diagnosed using the Gelman-Rubin test^59^,^60^. The posterior mean of individual effects was 0.653 with the credible interval ranging from 0.240 to 1.156 while observation-level variance was unidentifiable due to the binary nature of the outcome variable. Traces of the performed MCMCs validated convergence for the mixed model equation (MME) solutions, which was supported by the Gelman-Rubin diagnostic (multivariate scale reduction factor: 1.05).

Similarly, we analyzed the experimental part of the study using a Bayesian mixed effects Poisson model, using the count of interactions butterflyfishes exhibited with coral patches as response variable, while the experimental treatment (control, coral associated with *G. filamentosa*, corals associated with *G. polycystum*), the cue provided (visual or chemical), and the reliance on coral as a dietary item (obligate or opportunistic corallivores) served as fixed predictors. The higher order classification in obligate corallivores and opportunistic corallivores was chosen, as initial results indicated behavioral differences among the two groups and because species level data were too sparse due to the complete absence of some species from focal trials. Given the repeated use of patch-reef sets for visual and chemical trials, we fitted the patch-reef set to have a random effect. As for the observational data, complete separation in the dataset required the use of weakly informative priors on the fixed effects^58^. Non-informative priors inverse Wishart priors were used for the random effect and the residual variance, as observation-level variance is identifiable for Poisson models. Similar to the observational analyses, we ran three chains with 3,000,000 draws, a burn-in of 50,000 iterations and a thinning interval of 1,000 and validated chain convergence visually and analytically using the Gelman-Rubin test. For both models, we then predicted the model values and credible intervals on the data scale while marginalizing over the random effects. For the experimental model, we divided the predicted values by the number of individuals present during respective trials to standardize estimates as the number of interactions per fish per trial. All analyses were performed in *R*, using the packages *MCMCglmm*^57^ and *coda*^60^.

## Additional Information

**How to cite this article**: Brooker, R. M. *et al.* Cryptic effects of habitat declines: coral-associated fishes avoid coral-seaweed interactions due to visual and chemical cues. *Sci. Rep.* **6**, 18842; doi: 10.1038/srep18842 (2016).

## Supplementary Material

Supplementary Information

## Figures and Tables

**Table 1:**
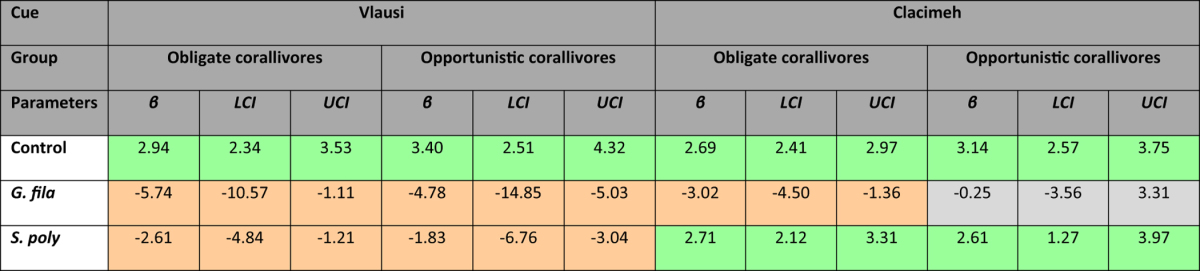
Model outputs from the Bayesian Mixed Model analyzing the response of butterflyfishes to a controlled patch reef experiment permitting foraging on corals associated with the algae *Galaxaura filamentosa* (“*G. fila.*”) or *Sargassum polycystum* (“*S. poly.*”), or control corals without algal association (“Control”).

Experimental setups included either a visual or a chemical cue of the algal association and data were analyzed for obligate and opportunistic corallivores. Provided model parameters include the posterior mean effect (“*β*”) as well as the lower (LCI) and upper (UCI) 95% credible intervals. Color shading indicates effect direction (red = negative, green = positive, grey = neutral). Trace plots of the MME solutions and the variance-covariance (VCV) solutions validated convergence of model chains and the Gelman-Rubin diagnostic performed on three independent chains confirmed convergence (multivariate scale reduction factors: MME = 1.01; VCV(set) = 1.01, VCV(units) = 1.00).
