# Supplementary material for: Cryptic effects of habitat declines: coral-associated fishes avoid coral-seaweed interactions due to visual and chemical cues
Source: Sci Rep. 2016 Jan 4;6:18842. doi: 10.1038/srep18842 (PMC4698739; doi:10.1038/srep18842)
Supplement: Supplementary Information [file srep18842-s1.doc]

**Supplementary Information:**

**Cryptic effects of habitat declines: coral-associated fishes avoid coral-seaweed interactions due to visual and chemical cues**

Authors: 1. Rohan M. Brooker^a, b^, 2. Simon J. Brandl^c, d, e^, 3. Danielle L. Dixson^a, b^

^a^School of Marine Science and Policy, University of Delaware, Lewes, DE 19958, USA

^b^School of Biology, Georgia Institute of Technology, Atlanta, GA 30318, USA

^c^ Tennenbaum Marine Observatories Network, Smithsonian Environmental Research Centre, Edgewater, MD, 21037, USA

^d^College of Tropical and Marine Science, James Cook University, Townsville, QLD 4811, Australia

^e^ARC Centre of Excellence for Coral Reef Studies, James Cook University, Townsville, QLD 4811, Australia

**Supplementary table S1:** Total number of observed interactions with coral by each species of *Chaetodon* butterflyfish during the survey of butterflyfish-coral-seaweed associations. Also shown is the observed probability that each butterflyfish would select seaweed-free coral and standard deviation.

| Species | n of interactions | Observed probability of selecting seaweed-free coral | SD |
| --- | --- | --- | --- |
| *Chaetodon auriga* | 518 | 0.918918919 | 0.27322345 |
| *Chaetodon bennetti* | 556 | 0.949640288 | 0.21888303 |
| *Chaetodon citrinellus* | 549 | 0.976320583 | 0.15218702 |
| *Chaetodon ephippium* | 681 | 0.953010279 | 0.21177238 |
| *Chaetodon lunula* | 670 | 0.964179104 | 0.18598222 |
| *Chaetodon ornatissimus* | 676 | 0.973372781 | 0.16111054 |
| *Chaetodon plebeius* | 576 | 0.96875 | 0.17414387 |
| *Chaetodon lunulatus* | 522 | 1 | 0 |
| *Chaetodon vagabundus* | 643 | 0.950233281 | 0.21763192 |
